# Prevalence and prognosis of myocardial scar in patients with known or suspected coronary artery disease and normal wall motion

**DOI:** 10.1186/1532-429X-13-2

**Published:** 2011-01-06

**Authors:** Rungroj Krittayaphong, Pairash Saiviroonporn, Thananya Boonyasirinant, Suthipol Udompunturak

**Affiliations:** 1 Division of Cardiology, Department of Medicine, Siriraj Hospital, Mahidol University, Bangkok, Thailand; 2 Department of Radiology, Siriraj Hospital, Mahidol University, Bangkok, Thailand; 3 Department of Research Promotion, Siriraj Hospital, Mahidol University, Bangkok, Thailand

## Abstract

**Background:**

Some patients may have normal wall motion after myocardial infarction. The aim of this study was to determine the prevalence and prognosis of patients with myocardial scar in the absence of abnormal wall motion. We studied patients with suspected or known coronary artery disease (CAD) who were referred for cardiovascular magnetic resonance (CMR) for the assessment of global and regional cardiac function and late gadolinium enhancement (LGE) and had normal left ventricular wall motion. Prognostic value was determined by the occurrence of hard endpoints (cardiac death and nonfatal myocardial infarction) and major adverse cardiac events (MACE) which also included hospitalization due to unstable angina or heart failure or life threatening ventricular arrhythmia.

**Results:**

A total 1148 patients (70.3%) were studied. LGE was detected in 104 patients (9.1%). Prevalence of LGE increased in patients with increased left ventricular mass. Average follow-up time was 955 ± 542 days. LGE was the strongest predictor for hard endpoints and MACE.

**Conclusion:**

LGE was detected in 9.1% of patients with suspected or known CAD and normal wall motion. LGE was the strongest predictor of significant cardiac events.

## Background

Assessment of structural heart disease or ventricular function has been recommended for patients presenting with signs or symptoms of heart disease such as dyspnea on exertion, heart failure [1], chest pain or angina [2], and acute coronary syndrome [3]. Structural heart disease such as valvular heart disease and global or regional ventricular function is usually evaluated by echocardiography [1-3]. Left ventricular ejection fraction (LVEF) is one of the most important indices of global left ventricular function and wall motion abnormality represents regional myocardial function. The prevalence of myocardial infarction by clinical history may be underestimated since approximately 20-40% of myocardial infarction may be unrecognized [4,5]. The prognostic importance of late gadolinium enhancement (LGE) has been reported in many groups of patients such as coronary artery disease (CAD) [6], non-ischemic cardiomyopathy [7] and diabetic patients [8]. It has been shown that the presence [6,8] and size [8] of myocardial scar and the presence of abnormal wall motion [8] had an impact on the prognosis of patients without clinical history of myocardial infarction. Little is known about the prevalence and prognosis of myocardial scar in patients with known or suspected CAD and normal wall motion.

Cardiovascular magnetic resonance (CMR) is considered the gold standard for the assessment of global ventricular function [9,10] and a good tool for the assessment of regional ventricular function [11]. It also provides the data concerning myocardial scar, most commonly related to myocardial infarction, by LGE technique. This technique has been proven to be very accurate, comparable to histopathology [12], and have better accuracy than single photon emission computed tomography [12,13], even in the setting of very small infarction [14], and it has also been shown to be highly reproducible [15].

The objectives of this study were 1) to determine prevalence of myocardial scar in patients with known or suspected CAD in the absence of abnormal wall motion and 2) to determine the prognostic value of myocardial scar in patients with known or suspected CAD in the absence of abnormal wall motion.

## Methods

### Study population

We studied patients who were referred for CMR from January 2002 to December 2007. Patients were referred for CMR due to clinical symptoms suspected to be related to CAD. Patients were included in this study if 1) known or suspected CAD who was referred for CMR for the evaluation of myocardial function and LGE 2) age more than 30 years and 3) normal left ventricular wall motion from CMR. Types of symptoms are shown in Table 1. Patients were excluded if any of the following criteria is presence: 1) had contraindication for CMR such as pacemaker or internal defibrillator implantation 2) history of myocardial infarction documented by standard criteria [16] 3) poor quality images for myocardial function or LGE 4) inability to complete CMR examination 5) history of revascularization 6) known disease that could cause LGE such as dilated cardiomyopathy [17], hypertrophic cardiomyopathy [18], myocarditis [19], cardiac amyloidosis [20] 7) clinically unstable conditions 8) need for urgent revascularization and 9) data unobtainable on clinical-follow-up. Since we excluded patients with history of myocardial infarction and history of revascularization, known CAD was defined as a history of at least 50% diameter stenosis of one or more major coronary arteries from coronary angiography who did not undergo revascularization and those with history of myocardial ischemia by nuclear study.

**Table 1:** Baseline characteristics of patients with and without LGE. Values are numbers (percentages) unless otherwise stated.

Characteristics	All N = 1148	LGE N = 104	No LGE N = 1044	P Value
Male	481 (41.9)	66 (63.5)	415 (39.8)	< 0.001
Mean (SD) age (years)	64.6 (11.3)	64.9 (12.5)	64.6 (11.2)	0.817
Smoking	148 (12.9)	19 (18.3)	129 (12.4)	0.086
Hypercholesterolemia	729 (63.5)	77 (74)	652 (62.5)	0.019
Diabetes mellitus	381 (33.2)	40 (38.5)	341 (32.7)	0.231
Hypertension	732 (63.8)	76 (73.1)	656 (62.8)	0.038
History of dyspnea on exertion	477 (41.6)	53 (51)	424 (40.6)	0.041
History of heart failure	23 (2)	2 (1.9)	21 (2)	0.951
Presence of chest pain	576 (50.2)	48 (46.2)	528 (50.6)	0.390
Medication				
-Beta blockers	487 (42.4)	48 (46.2)	439 (42)	0.419
-Calcium channel blockers	288 (25.1)	21 (20.2)	267 (25.6)	0.227
-Nitrates	284 (24.7)	27 (26)	257 (24.6)	0.762
-Aspirin/clopidogrel	600 (52.3)	60 (57.7)	540 (51.7)	0.245
-ACEI/ARB	334 (29.1)	40 (38.5)	294 (28.2)	0.027
-Statins	541 (47.1)	59 (56.7)	482 (46.2)	0.040
MI by ECG	89 (7.8)	17 (18.1)	72 (7.4)	< 0.001
CMR variables in mean (SD)				
-LVEF (%)	71.0 (9.1)	69.8 (10.7)	71.1 (9.0)	0.255
-LVEDVI (ml/m^2^)	60.6 (19.6)	62.4 (22.0)	60.4 (19.4)	0.320
-LVESVI (ml/m^2^)	18.6 (14.1)	20.5 (17.7)	18.4 (13.7)	0.258
-LVMASSI (g/m^2^)	48.8 (17.3)	63.0 (31.7)	47.3 (14.5)	< 0.001

LGE = late gadolinium enhancement, MI = myocardial infarction, CMR = cardiovascular magnetic resonance, ACEI = angiotensin converting enzyme inhibitors, ARB = angiotensin receptor blockers, LVEF = left ventricular ejection fraction, LVEDVI = left ventricular end-diastolic volume index, LVESVI = left ventricular end-systolic volume index, LVMASSI = left ventricular mass index

This study was approved by the Ethics committee of Siriraj Hospital. All patients provided written informed consent.

### CMR protocol

All patients underwent CMR which included functional study and assessment of LGE using a 1.5 Tesla Gyroscan NT Philips scanner (Philip Medical System, Best, the Netherlands). After scout images, functional study was performed with a steady-state free-precession technique in horizontal long axis view, 2-chamber view, 4-chamber view and multiple slice short axis view. LGE was performed in 3-D fashion 7-10 minute after the injection of gadolinium 0.2 mmol/kg. Multiple short-axis slices at the same level as the functional study, 2-chamber and 4-chamber view were acquired for LGE.

Parameters for functional images were as follows: repetition time/echo time/number of excitations = 3.7/1.8/2, 390 × 312 mm field of view, 256 × 240 matrix, 1.52 × 1.21 reconstruction pixel, 8 mm slice thickness, 70 degree flip angle. Typical temporal resolution was 25-30 ms. The LGE images were acquired with the use of 3 D segmented-gradient-echo inversion-recovery sequence with echo time 1.25, repetition time 4.1, 15 degree flip angle, 303 × 384 mm field of view, 240 × 256 matrix, in-plane resolution 1.26 × 1.5 mm, slice thickness 8 mm and 1.5 Sensitivity encoding factor. The whole study for each patient took approximately 40 minutes. Patients had ECG performed on the same day before undergoing CMR. Myocardial infarction by ECG was defined by the standard criteria [16].

### Analysis of CMR

The analysis was performed on the ViewForum workstation (Philip Medical System, Best, the Netherlands). Functional CMR data were analyzed for volume, mass and ejection fraction of the left ventricle. The software made an automatic detection of the endocardial and epicardial border of the left ventricle during diastole and endocardial detection for images during systole. Manual adjustment was performed by an experienced technician. The software then made calculations for left ventricular end-diastolic volume (LVEDV), left ventricular end-systolic volume (LVESV), left ventricular mass (LVMASS), and LVEF. Calculation of indices of LVEDV (LVEDVI), LVESV (LVESVI) and LVMASS (LVMASSI) was performed to adjust for body surface area. Intra- and interobserver variability presented as percentages of the mean of 2 repeated measurements averaged ± standard deviations were 3 ± 4% and 4 ± 4% for LVEDV, 4 ± 5% and 6 ± 6% for LVESV and 3 ± 4% and 5 ± 5% for LVMASS.

LGE was analyzed by visual estimation for the presence or absence of LGE and quantification of LGE. LGE was graded segmental extent of LGE as follows: 0 = no scar, 1 = 1-25%, 2 = 26-50%, 3 = 51-75%, and 4 = 76-100% of LGE areas in comparison to segmental myocardial areas. Analysis of myocardial segments was based on 17-segment models [21] with the exclusion of segment 17 from analysis. Slices was classified as basal segments when tips of mitral valve is visualized, mid-cavity segments when papillary muscles are visualized and apical segments when they are beyond papillary muscles but before the cavity ends [21]. Each of the basal and mid-cavity slices was divided into 6 segments and each of the apical slices was divided into 4 segments. Total scar size was derived from the summation of scar grading of all segments, divided by 4 times the total myocardial segments and calculated as percentages of scar in comparison with myocardium [12].

Analysis of wall motion was performed by visual assessment. Wall motion and LGE was assessed by 2 readers independently of the history. Disagreement was solved by a 3^rd ^reader. Regional wall motion was analyzed by the use of the 17-segment model proposed by the American Heart Association [21]. Wall motion of each myocardial segment was recorded as 5-grade system as followed: 1 = normal, 2 = hypokinesia, 3 = akinesia, or 4 = dyskinesia. Intra- and interobserver agreement for the presence of a LGE area in our center were k = 0.94, p <0.001 and k = 0.97, p <0.001 respectively using a signal intensity threshold of more than 2 standard deviations above the signal intensity of a remote myocardial region. The cut off of 2 standard deviations has been used in many previous studies [6-8]. Visual detection for LGE has been shown to be in a good agreement with the conventional technique with k = 0.952, p < 0.001 [22]. For the presence of abnormal wall motion, intra- and interobserver agreement were k = 0.95, p <0.001 and k = 0.93, p <0.001 respectively

### Clinical follow-up

Primary outcomes include both hard cardiac events and major adverse cardiac events (MACE). Cardiac death and myocardial infarction were considered as hard cardiac events. MACE includes hard cardiac events and hospitalization due to unstable angina, heart failure or life-threatening ventricular arrhythmia. Assessment of cardiac events was performed by the review of medical records and telephone interview followed by document confirmation.

### Statistical analysis

Data were described as mean ± standard deviation (SD) for continuous data and count (percentages) for categorical data. Independent-samples T-test was used for the comparison of continuous data. Comparisons of categorical data were made by the Chi-square test or Fisher exact test wherever appropriate. Cox-regression analysis was used for the assessment of predictors for clinical outcomes and was described as hazard ratio and 95% confidence interval (CI). Kaplan-Meier analysis with log-rank test was used for survival analysis with comparison of survival data between groups. A p-value of ≤ 0.05 was considered significant.

## Results

We excluded 248 patients with a history of revascularization, 252 patients with a history of myocardial infarction, 18 patients who were unable to complete CMR examination, 8 patients with inadequate image quality, 4 patients with pacemakers, 5 patients with unstable clinical conditions, 14 patients unobtainable clinical follow-up data, 10 patients with mid-wall or patchy scar likely to be non-ischemia cardiomyopathy scar, 3 patients with scar at the insertion site of right ventricular free-wall likely to be related to hypertrophic cardiomyopathy, 2 patients with subepicardial scar likely to be related to myocarditis, and 1 patient with diffuse LGE likely to be related to amyloidosis. After the exclusion, total of 1148 patients with normal left ventricular wall motion were included in this study. Average age was 64.6 ± 11.3 years. LGE was detected in 104 patients (9.1%). Table 1 shows baseline characteristics of patients with and without LGE. The following factors were associated with LGE: male gender, hypercholesterolemia, hypertension, a history of dyspnea on exertion, use of certain medications such as angiotensin converting enzyme inhibitor (ACEI) or angiotensin receptor blocker (ARB), and statin. There were 132 patients (11.5%) who underwent coronary angiogram before CMR study. The results of coronary angiogram showed single vessel disease in 35 (27%), 2-vessel disease in 32 (24%), 3-vessel disease in 29 (22%) and no significant CAD in 36 patients (27%).

**Figure 1:**
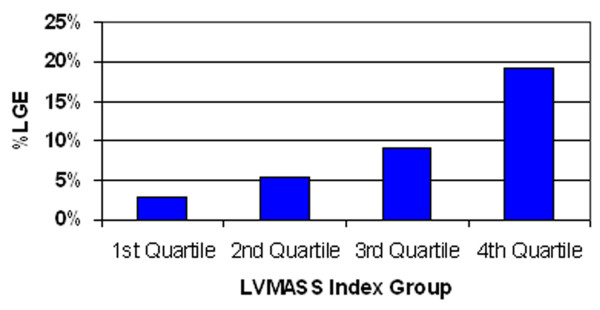
**Percentages of LGE stratified by quartiles of LVMASS index**.

Comparisons of CMR functional parameters were performed (Table 1). There was no significant difference in LVEF between patients with and without LGE. LVMASS index was significantly greater in patients with LGE in comparison to those without LGE. We further explored the relation between increased LVMASS index and the presence of LGE by dividing LVMASS index into 4 quartile groups. The prevalence of LGE was only 2.8% in the lowest quartile of LVMASS index group. The prevalence increased to 5.2%, 9.1% and 19.2% in the 2^nd^, 3^rd ^and highest quartile respectively (Figure 1).

**Figure 2:**
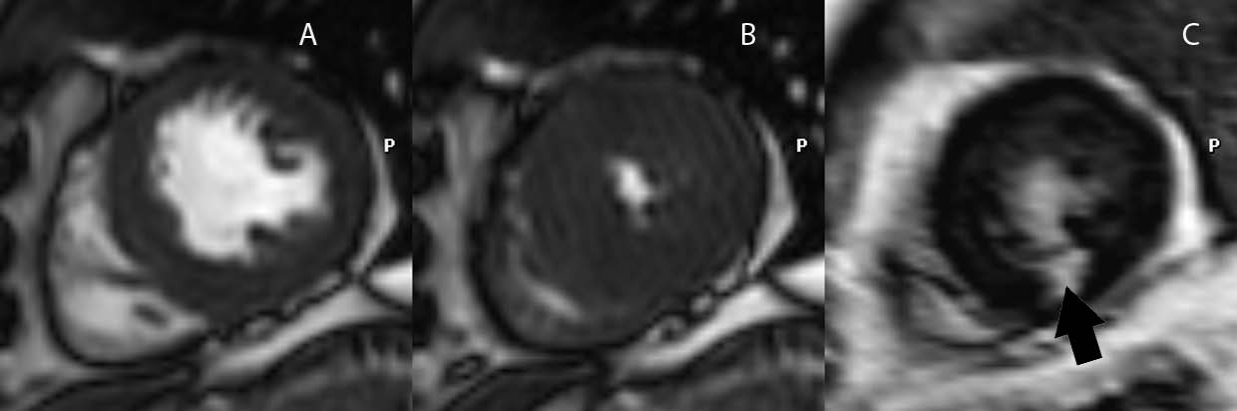
**Functional images at diastole (A), systole (B) and LGE images (C) of a patient with LGE at inferior wall (black arrow), normal wall motion and increased left ventricular mass index**.

Coronary angiography data after CMR of those who had LGE was explored to confirm the underlying CAD in this group. Among 104 patients with LGE, 48 (46.2%) underwent coronary angiography, 12 had single vessel disease, 15 had 2-vessel disease, 20 had 3-vessel disease, and only 1 patient had no significant CAD. Among those who underwent coronary angiography, 24 were in the highest quartile of LVMASS index, 13 in the 3^rd ^quartile, 4 in the 2^nd ^quartile and 7 in the lowest quartile. Figure 2 shows example of a patient with increased LVMASS index (3^rd ^quartile group) had LGE without abnormal wall motion. Among patients without LGE, coronary angiogram was performed in 145 patients (13.9%) which demonstrated single vessel disease in 39, 2-vessel disease in 30, 3-vessel disease in 30, and no significant CAD in 46 patients.

### Predictors for cardiac events

**Figure 3:**
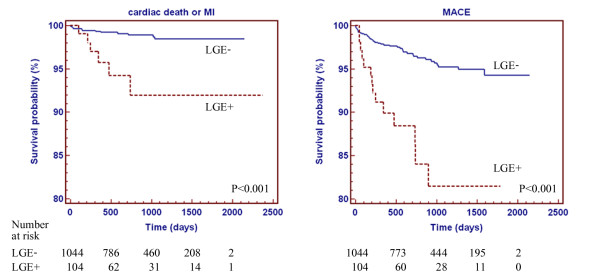
**Kaplan-Meier graph of LGE and occurrence of hard cardiac events (left) and MACE (right)**.

During an average follow-up time of 955 ± 542 days, 18 patients (1.6%) had hard cardiac events and 54 (4.7%) had MACE. The frequency of cardiac events is shown in Table 2. Univariate predictors and multivariable analysis for hard cardiac events and MACE were analyzed (Table 3 and 4). LGE is the only significant predictor for MACE and strongest predictor for hard cardiac event followed by myocardial infarction by ECG. However, as in Table 1 chance of having ECG evidence of myocardial infarction was small in patients with LGE. Besides, myocardial infarction diagnosed by ECG had a significant chance of false positive as it was noted in 7.4% of patients without LGE. Overall sensitivity, specificity, positive predictive value and negative predictive value of ECG evidence of myocardial infarction for the detection of LGE were 18.1%, 92.6%, 19.1%, and 92.1% respectively. Kaplan-Meier graph with log rank test of LGE as the predictor for hard cardiac events and MACE is shown in Figure 3. Since there may be a possibility that some patients may undergo coronary angiography or revascularization related to the results of CMR and had cardiac events caused by the procedures, we looked at the rate of cardiac events related to the procedures in both groups within 6 months after the CMR. Revascularization was performed in 28 patients (26.9%) who had LGE and 79 patients who had no LGE (7.6%). There were a total of 5 patients who had MACE related to revascularization within 6 months after CMR: 1 in LGE group (0.96%) and 4 in non-LGE group (0.38%) which had no statistical significance (p = 0.379).

**Table 2:** Summary of clinical events during follow-up. Values are numbers (percentages).

Clinical events	Number (%)
Total death	32 (2.8)
Cardiac death	5 (0.4)
Myocardial infarction	13 (1.1)
Hospitalization due to heart failure	21 (1.8)
Hospitalization due to unstable angina	26 (2.3)
Life-threatening ventricular arrhythmia	1 (0.1)
Coronary angiogram	172 (15)
-no significant coronary artery disease	48 (27.9)
-significant coronary artery disease	124 (72.1)
-single vessel disease	44 (35.5)
-double vessel disease	38 (30.7)
-triple vessel disease	42 (33.8)
Percutaneous coronary intervention	77 (6.7)
Coronary bypass surgery	30 (2.6)

**Table 3:** Univariate predictors of hard endpoints and MACE

Clinical characteristics	Cardiac Death or Nonfatal MI (N = 18-1.6%)	P Value	MACE (N = 54 - 4.7%)	P Value
Male	1.42 (0.56-23.57)	0.460	1.13 (0.66-1.93)	0.655
Body mass index > 25 kg/m^2^	0.25 (0.08-0.77)	0.016	0.72 (0.42-1.22)	0.220
Age (per 10 year increment)	0.94 (0.63-1.40)	0.756	1.15 (0.90-1.48)	0.256
Smoking	0.48 (0.06-3.61)	0.475	0.67 (0.24-1.85)	0.437
Hypercholesterolemia	0.95 (0.37-2.44)	0.909	1.22 (0.69-2.15)	0.487
Diabetes mellitus	1.058 (0.62-4.00)	0.335	2.01 (1.18-3.42)	0.010
Hypertension	0.73 (0.29-1.86)	0.514	1.10 (0.63-1.93)	0.730
Prior chest pain	0.30 (0.10-0.90)	0.032	0.84 (0.49-1.44)	0.523
Prior dyspnea	0.95 (0.37-2.46)	0.922	0.82 (0.47-1.43)	0.473
Prior heart failure	1.58 (0.56-5.85)	0.235	2.18 (0.53-8.96)	0.279
**Medication**				
-Beta blockers	0.38 (0.13-1.17)	0.092	0.93 (0.54-1.61)	0.805
-Calcium antagonist	0.19 (0.42-3.34)	0.742	1.20 (0.66-2.17)	0.556
-Nitrates	0.90 (0.30-2.72)	0.845	1.01 (0.54-1.88)	0.986
-Aspirin/clopidogrel	0.60 (0.23-1.52)	0.274	1.02 (0.60-1.73)	0.954
-ACEI/ARB	0.70 (0.23-2.13)	0.531	0.87 (0.47-1.5)	0.644
-Statins	0.60 (0.22-1.59)	0.301	0.71 (0.41-1.23)	0.216
**MI by ECG**	7.02 (2.20-22.38)	0.001	1.65 (0.59-4.60)	0.340
**CMR variables**				
-presence of LGE	5.74 (2.15-15.33)	< 0.001	4.12 (2.23-7.57)	< 0.001
-LVEF (per 10% decrement)	1.39 (0.90-2.14)	0.135	1.19 (0.91-1.56)	0.208
-LVEDVI	1.007 (1.000-1.014)	0.041	1.006 (1.000-1.011)	0.038
-LVESVI	1.012 (1.001-1.024)	0.040	1.009 (1.000-1.019)	0.054
-LVMASS index (per quartile)	2.57 (1.36-6.45)	< 0.001	2.11 (1.27-5.56)	< 0.001

MI = myocardial infarction, MACE = major adverse cardiac event, ACEI = angiotensin converting enzyme inhibitors, ARB = angiotensin receptor blockers, CMR = cardiovascular magnetic resonance, LGE = late gadolinium enhancement, LVEF = left ventricular ejection fraction, LVEDVI = left ventricular end-diastolic volume index, LVESVI = left ventricular end-systolic volume index, LVMASSI = left ventricular mass index

**Table 4:** Multivariable associations for hard endpoints and MACE

	HR (95% CI)	P Value
**Cardiac death or nonfatal MI**		
-LGE	5.43 (1.71-17.25)	0.004
-MI by ECG	5.14 (1.62-16.29)	0.005
**MACE**		
-LGE	3.92 (1.98-7.76)	< 0.001

MACE = major adverse cardiac event, HR = hazard ratio, CI = confidence interval, MI = myocardial infarction, LGE = late gadolinium enhancement

Additional analysis was performed for the assessment of the prognostic importance of the extent of LGE. By using the median size of LGE of 6.25% of myocardium, we graded the extent of LGE into 3 groups: no LGE, LGE ≤ median and LGE > median. Hazard ratios and 95%CI for hard cardiac events and MACE for patients with LGE ≤ median were 2.14 (0.28-16.53), p = 0.467 and 3.36 (1.32-8.55), p = 0.011 compared to those without LGE. Multivariable analysis showed that patients with LGE ≤ median had no significant increase in risk of hard events but had a significant increase in risk of MACE (hazard ratio 3.28, 95%CI 1.29-8.37, p = 0.013) compared to those without LGE. For patients with LGE > median, hazard ratios for hard events and MACE were 8.59 (3.23-24.37), p < 0.001 and 4.69 (2.28-9.67), p < 0.001 compared to those without LGE. Multivariable analysis showed that patients with LGE > median had a significant increase in risk of hard events (hazard ratio 7.96, 95%CI 2.34-27.09, p = 0.001) and MACE (hazard ratio 4.51, 95%CI 2.19-9.30, p < 0.001) compared to those without LGE.

## Discussion

The results of our study showed that in patients with known or suspected CAD and normal wall motion, 9.1% had LGE. Patients with LGE had an increased risk of cardiovascular events compared to those without. In fact, LGE is the strongest predictor of cardiac events.

Echocardiography is an investigation for the evaluation of evidence of structural heart disease in patients with sign or symptoms of heart disease [1-3]. CMR, although of limited availability and more expensive, can assess evidence of structural heart disease to a greater extent than echocardiography [17,18,20]. Although echocardiography may have a better temporal resolution than CMR, the overall image quality of CMR for the assessment of regional wall motion is usually better than echocardiography (11, 23). In comparison with echocardiography, CMR has a better tissue contrast and better border definition thereby better endocardial detection. Image quality of echocardiography may also be compromised due to a poor acquisition window. CMR has been shown to be very accurate for the detection of myocardial scar [12] which may be related to a better image quality of CMR in comparison to echocardiography [9]. A previous study [5] showed that patients with unrecognized myocardial infarction diagnosed from ECG had regional wall motion abnormality, from echocardiogram, in 13% compared to 42% in those with recognized myocardial infarction. This may be due to the limited accuracy of ECG criteria for the diagnosis of myocardial infarction [4].

We showed that, among patients with normal wall motion, the prevalence of LGE increased in patients with high LVMASS index with the prevalence up to 19% in the highest quartile of LVMASS index. This finding is consistent with a previous report [5] which showed an increased LVMASS index from echocardiogram in patients with unrecognized myocardial infarction.

Compared to earlier report [6] on the prognostic importance of LGE in patients with known or suspected CAD, we focused on patients with normal wall motion which has not been reported before. We also had a larger number of patients with normal wall motion. Although they reported the findings of LGE and abnormal wall motion, the number of patients with normal wall motion in their study was small and cannot provide the prognostic data in this group. Calculation from the data that they reported, the prevalence of LGE in patients with normal wall motion was 7% which is slightly less than the prevalence in our study.

The results of this study on the prevalence of LGE and its prognostic value can be applied to patients who were referred for CMR with normal left ventricular wall motion without known cause of LGE. This is the reason that we excluded patients with history of myocardial infarction and other known causes of LGE including those with history of revascularization which may have a significant number of procedure-related LGE which was detected by CMR in up to 64% [14].

There are some limitations of this study. Firstly, although we exclude hypertrophic cardiomyopathy and many conditions that might cause LGE, we did not exclude left ventricular hypertrophy or aortic stenosis which has been shown to have an association with LGE [24]. However, in this study, we did not have any patients with severe aortic stenosis and LGE was an independent predictor after the adjustment of LVMASS. Secondly, the detection of abnormal wall motion was performed by visual assessment without the use of quantitative analysis although intra- and inter-observer agreements for wall motion assessment in our study were excellent as shown earlier. Lastly, this study did not analyze the influence of inducible wall motion abnormality or perfusion abnormality that might also have an effect on clinical outcome due to inadequate number of patients.

## Conclusion

This is the first study that demonstrated the prognostic importance of LGE in patients with normal wall motion. The prognostic value of LGE in this patient population was even more confirmed by the quantitative analysis of LGE. Therefore, clinicians cannot assume that patients without wall motion abnormality have no evidence of structural heart disease and have a good prognosis. Although myocardial infarction by ECG was also an independent predictor for hard endpoints, LGE was the strongest predictor of hard endpoints and MACE.

## List of abbreviations

ACEI: angiotensin converting enzyme inhibitor; ARB: angiotensin receptor blocker; CAD: coronary artery disease; CI: confidence interval; CMR: cardiovascular magnetic resonance; HR: hazard ratio; LGE: late gadolinium enhancement; LVEDV: left ventricular end-diastolic volume; LVEDVI: left ventricular end-diastolic volume index; LVEF: left ventricular ejection fraction; LVMASS: left ventricular mass; LVMASSI: left ventricular mass index; LVESV: left ventricular end-systolic volume; LVESVI: left ventricular end-systolic volume index; MACE: major adverse cardiac event; MI: myocardial infarction, SD: standard deviation

## Competing interests

The authors declare that they have no competing interests.

## Authors' contributions

RK study design, data interpretation, writing manuscript, PS data acquisition and interpretation, revise manuscript, TB data interpretation, revise manuscript, SU data analysis and interpretation, revise manuscript. All authors approved the final version of the manuscript.
